# Primary cutaneous cryptococcosis: the importance of early diagnosis^☆^^☆☆^

**DOI:** 10.1016/j.abd.2020.12.004

**Published:** 2021-05-24

**Authors:** Cacilda da Silva Souza, Maria Hideko Takada, Marcela Vendruscolo Ambiel, Viviane Tiemi Nakai

**Affiliations:** Division of Dermatology, Department of Internal Medicine, Faculdade de Medicina de Ribeirão Preto, Universidade de São Paulo, Ribeirão Preto, São Paulo, Brazil

**Keywords:** Cryptococcosis, Fungal diseases, Skin, Skin diseases

## Abstract

The species of the *Cryptococcus neoformans* complex show different epidemiological patterns in the infection of immunosuppressed or immunocompetent individuals, and a common tropism peculiarity for the central nervous system. Primary cutaneous cryptococcosis is a rare clinical entity, with manifestations that are initially restricted to the skin through fungal inoculation, and the absence of systemic disease. The authors report in the present study the case of a 61-year-old immunocompetent man, with a rapidly evolving mucoid tumor on abrasions in contact with bird droppings on the forearm. The early identification of the polymorphic skin manifestations and treatment are crucial for the favorable prognosis of the infection, which can be life-threatening.

In cryptococcosis, a fungal infection caused by encapsulated yeasts, the species *Cryptococcus neoformans var.grubii* and *C. neoformans var. neoformans* are associated with immunocompromised or immunocompetent hosts, and areas of bird excrement grouping; whereas the species *Cryptococcus gattii*, relates to immunocompetent hosts and plant residues (*Eucalyptus camaldulensis*) from tropical and subtropical areas.^1^, ^2^, ^3^

After yeast inhalation, pulmonary involvement associated with meningoencephalitis can occur in a significant number of cases, with cutaneous manifestations occurring in 10% to 15% of them, due to the hematogenous dissemination of the infection. Primary cutaneous cryptococcosis (PCC) is a rare entity, initially restricted to the skin, caused by direct fungal inoculation, with no signs of systemic disease.^1^, ^2^, ^3^, ^4^ The primary or secondary cutaneous condition is polymorphic (papules, purpura, vesicles/blisters, pustules, nodules, tumors, ulcerations, necrotizing panniculitis/cellulite, abscesses, acne-like lesions and molluscum contagiosum-like lesions), and can delay the diagnosis and lead to unfavorable outcomes.^1^, ^2^, ^3^, ^4^, ^5^, ^6^, ^7^

The mortality, estimated at 10% in developed countries, can rise fourfold in countries such as Thailand. Although rare and with a favorable evolution, PCC can be life-threatening, particularly in patients with underlying diseases or immunocompromised patients, given the possibility of dissemination and central nervous system (CNS) involvement.^1^, ^2^, ^3^, ^6^

## Case report

**Figure 1:**
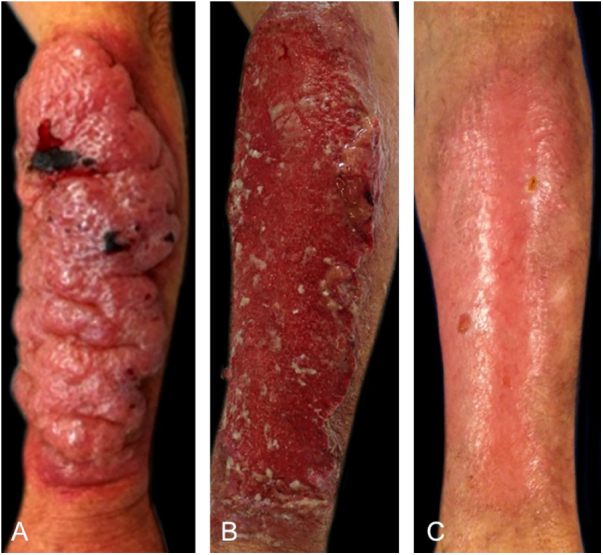
(A) Friable tumor with a mucoid aspect; (B) Post-detachment of the mucoid and necrotic plaque; (C) Healing at 120 days.

**Figure 2:**
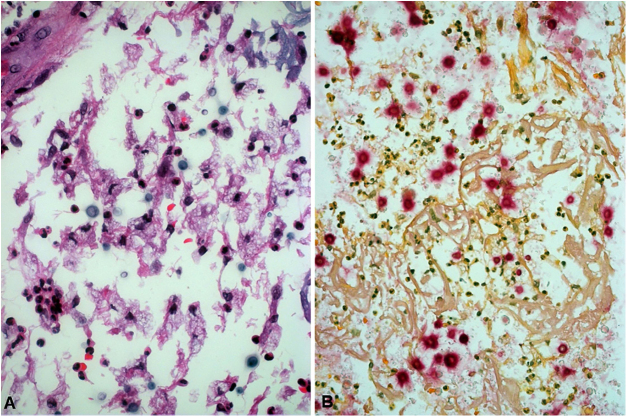
(A) Rounded structures with Grocott-Gomori silver methenamine staining; (B) and with Mayer's mucicarmine staining.

A 61-year-old male reported that 30 days before, a pruritic erythematous papule had appeared on his left forearm, which quickly progressed to a painful, friable and mucoid-like tumor (Fig. 1A). He had no fever, systemic complaints or lymph node enlargement, but the presence of excoriation on the forearms due to keeping and caring of birds. The PCC hypothesis and treatment with antifungal drugs were based on identification of yeast-like structures observed in the direct examination, that were later stained by Grocott-Gomori (Fig. 2A) and Mayer's mucicarmin stain (Fig. 2B) at the histopathological study, and identified by culture as *Cryptococcus neoformans*.

Serology study, including latex test for cryptococcosis and ELISA for HIV infection were negative; chest radiography and CSF analysis were normal. The treatment with intravenous fluconazole (400 mg daily), for 30 days, promoted the detachment of the mucoid and necrotic plaque (Fig. 1B), which was followed by oral use for 11 months with total re-epithelialization (Fig. 1C).

The PCC diagnosis criteria include the restriction to cutaneous manifestations with no evidence of systemic disease and positive culture for *Cryptococcus spp*.; and, additionally, single lesion or confined to one uncovered body area (limbs or face), previous traumas, abrasions, pre-existing skin ulcerations or lesions at the infection site and exposure to the contaminated source.^2^, ^4^

In the review of 11 cases of PCC in immunocompetent hosts, published in Brazil, older age was observed (mean of 70.91 years), as well as a predominance of male sex (81.82%), with previous trauma and/or exposure of the lesion site to contaminated sources (63.63%), and mean time of 62.14 days (15 to 210 days) until diagnosis. They shared forearm involvement, ulceration on plaques or nodules, shiny, mucoid or gelatinous nodules and/or tumors; pruritus at the start (three cases), pain and necrosis (two cases), cure for the majority of patients, but one death in a patient with cirrhosis and alcohol abuse.^4^, ^5^, ^6^, ^7^, ^8^, ^9^, ^10^

The anatomical site and the host's immune status are the treatment-defining factors. For the management of less common manifestations in the HIV-negative population with a single infection site, with no evidence of CNS involvement, fungemia or immunosuppression, oral fluconazole (6 mg/kg/daily) for 6 to 12 months has been recommended.^3^

Considering that the cutaneous manifestation may precede the disseminated infection, its identification and early therapeutic interventions are crucial for reducing unfavorable outcomes.

## Financial support

None declared.

## Authors’ contributions

Cacilda da Silva Souza: Design and planning of the study; collection, analysis, and interpretation of data; intellectual participation in the propaedeutic and/or therapeutic conduct of the studied case; critical review of the literature; drafting and editing of the manuscript.

Maria Hideko Takada: Approval of the final version of the manuscript; intellectual participation in the propaedeutic and/or therapeutic conduct of the studied case; critical review of the manuscript.

Marcela Vendruscolo Ambiel: Approval of the final version of the manuscript; intellectual participation in the propaedeutic and/or therapeutic conduct of the studied case; critical review of the literature; critical review of the manuscript.

Viviane Tiemi Nakai: Approval of the final version of the manuscript; collection, analysis, and interpretation of data; critical review of the literature; critical review of the manuscript.

## Conflicts of interest

None declared.
